# Secular trend in age at menarche among Indian women

**DOI:** 10.1038/s41598-024-55657-7

**Published:** 2024-03-05

**Authors:** Trupti Meher, Harihar Sahoo

**Affiliations:** International Institute for Population Sciences, Mumbai, India

**Keywords:** Reproductive biology, Biomarkers

## Abstract

Age at menarche is not only a parameter that signifies biological characteristics for women but is also considered as an indicator to measure the quality of life of a population. Moreover, menarche has significant implications on women’s health and information about menarcheal age is crucial for health policymakers. However, little is known about the trends in menarcheal age in India. Thus, in order to fill this research gap, the present study aimed to explore the age at menarche, its trend and regional heterogeneity among Indian women. A birth cohort approach was used by polling data from the 1st (1992–93), 4th (2015–16) and 5th (2019–21) rounds of NFHS. Descriptive statistics and bivariate analyses were performed to present the distribution of age at menarche and mean age at menarche across birth cohorts and each category of covariates. A multiple linear regression model was fitted to examine the trend in age at menarche and further to investigate the association of covariates with menarcheal age among Indian women. The analysis demonstrated that a majority of women (66.2%) attained menarche between the ages of 13–14 years. Moreover, about 17.2% of women experienced an early age at menarche, whereas 16.7% of women had a late age at menarche. The mean age at menarche for the sampled women was 13.49 years. The analysis also observed a secular declining trend in menarcheal age among Indian women and a significant variation in the mean age at menarche across birth cohorts. It also highlighted significant socio-economic patterning in menarcheal age among women.

## Introduction

A girl child’s life is marked by various milestones as she matures into a woman and becomes capable of reproducing and menarche is the first key event in this sexual development^1^. It is a unique biological marker that signifies the transition of a young girl from childhood to womanhood^2,3^. Age at menarche is not only a parameter that signifies biological characteristics for women but also considered as an indicator to measure the quality of life of a population^4^. In the diagnosis of delayed puberty, pathological and hormonal disorders, the menarcheal age is very crucial. Therefore, the health status of the female depends on her age at menarche.

This crucial developmental milestone in women has been observed to differ considerably throughout the world. A normal onset of menarche is considered to occur between the ages of 11–15 years^5–7^. However, huge spatial variations in age at menarche have been reported both between and within sub-national populations. The mean age at menarche in developed countries is lower than in developing countries. (13.05 years in France, 13.3 years in the United Kingdom, and 12.8 years in the United States, while it is 13.5 years in Sri Lanka, 15.8 years in Bangladesh, and 16.2 years in Nepal)^8^. Nevertheless, over the past four decades, the mean age at menarche reportedly varied from 12 to 16 years across various subgroups of Indian women^6,9–12^. However, most of the studies that reported menarcheal age above 13 years were conducted during 1980–2000^9–11^, while other studies that reported menarcheal age below 13 years were mostly conducted after 2000^6,12^.

Evidence from a number of studies from various countries has pointed towards the secular decline in the age of onset of menarche over the past few decades^13–15^. The European Prospective Investigation into Cancer & Nutrition (EPIC) study has found a decrease in average menarcheal age among female participants born from 1912 to 1964 in nine European countries^16^. Several other literatures have also documented a trend towards earlier menarche in France^17^, Israel^18^ and the USA^19^. However, Wahab et al. (2018)^20^, in their systematic review on declining age at menarche, have found that in higher-income countries, the lowering in mean age at menarche is earlier than the middle and lower-income countries. While the observed reduction in menarcheal age has leveled off in many industrialized North American and European nations, it has continued to fall in some developing nations^21–23^. Furthermore, a decreasing trend of age at menarche has been reported by most Indian studies^24,25^. Nevertheless, according to Bagga and Kulkarni (2000)^25^, the lowering of age at menarche in India was at an average rate of about 6 months per decade as compared to 3–4 months in some countries of Europe, North America. However, Pathak et al. (2014)^26^ have established a reduction of nearly one month per decade using the Indian Human Development Survey (IHDS) data.

Menarche is an important part of the complicated process of maturation. Therefore, the menarcheal onset cannot be traced to a single factor since the factors that influence the menarcheal age are interconnected^27,28^. Menarcheal age is known to be impacted by genetics, although the particular genetic drivers remain mostly unknown. Various studies from different regions of the world have shown that an acceleration of sexual maturation and physical growth follows a major improvement in socio-economic conditions. According to researchers like Tarannum et al. (2018)^6^, Rokade and Mane (2009)^12^, Wronka and Pawlińska-Chmara (2005)^29^, factor like socioeconomic status plays a vital role in determining the menarcheal age of girls. Socioeconomic factors such as wealth status, family size, place of residence, educational level of parents may also influence the age at onset of menstruation^26,29,30^.

The age at menarche, which is regarded as a marker of female reproductive maturity, has significant implications on women’s health^6^. Information about menarcheal age is crucial for health policymakers, particularly when it comes to providing health services and menstrual health education to schoolgirls^31^. However, previous studies on age at menarche in India are confined to limited geographic context^10–12^. Besides, in most of the previous studies, the units of analysis have been school girls, adolescents and sports women from a specific localized area^6,9^, making it difficult to draw any comparisons of age at menarche in India or in its any regional dimensions. Also, little is known about the trends in menarcheal age in the country. Thus, in order to fill this research gap, it is important to explore the trend and regional heterogeneity in the age at menarche in India using nationally representative survey data. Therefore, in the current study an attempt was made to determine the mean age at menarche and the prevalence of early, ideal and late menarche among Indian women. In addition, the study also focused on evaluating the trend and variations in age at menarche in the country.

## Results

### Distribution of women according to age at menarche

**Figure 1:**
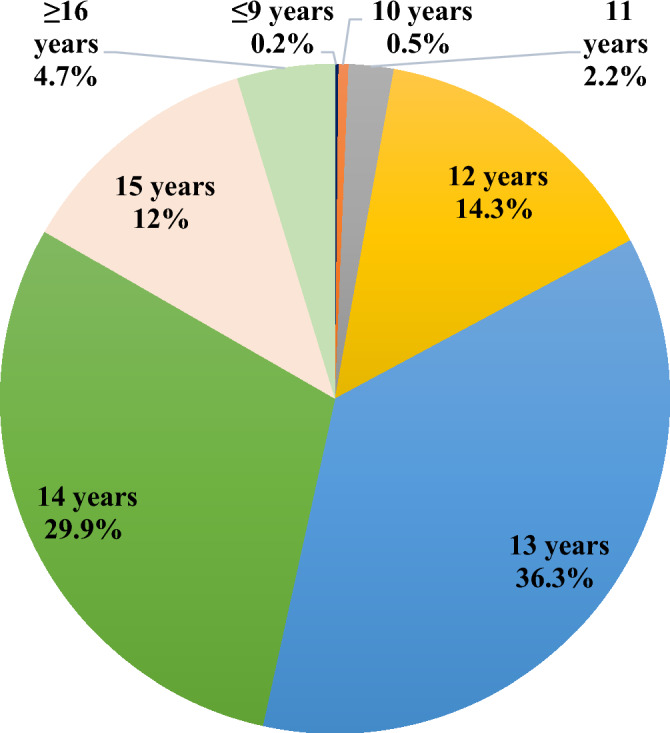
Distribution of women according to age at menarche.

Figure 1 shows the distribution of women according to age at menarche. As per the figure, the majority of women had attained menarche at the age of 13 years (36.3 years) and 14 years (29.9 years). Moreover, only 0.2 percent of women reported menarche at 7–9 years of age and 0.5 percent attained menarche at the age of 10 years. Furthermore, nearly 14.3 percent of studied women reported experiencing menarche at the age of 12 years and 12 percent experienced it at the age of 15 years. Nevertheless, 4.7 percent of women attained menarche at a later age of 16 and above.

**Figure 2:**
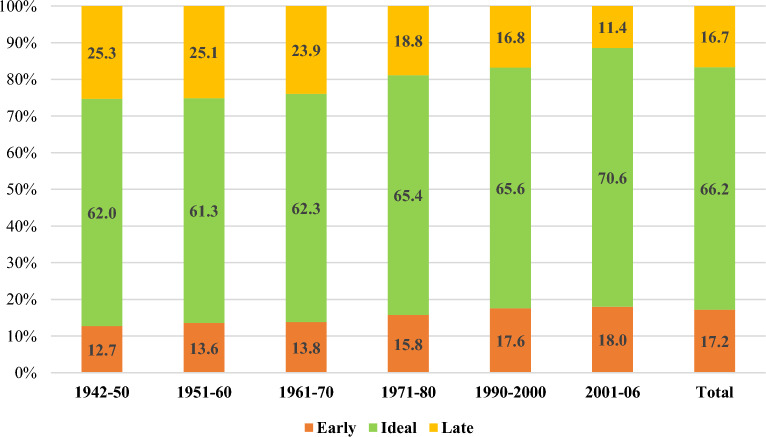
Distribution of age at menarche by birth cohort.

Figure 2 depicts the percentage distribution of age at menarche by birth cohort. The data demonstrated that a majority of women (66.2%) attained menarche between the ages of 13–14 years. Moreover, about 17.2% of women experienced an early age at menarche, whereas 16.7% of women had a late age at menarche. Furthermore, early age at menarche showed an increasing trend over the years. On the other hand, the percentage of women experiencing late age at menarche decreased substantially from 25.3 to 11.4% among women born during the period 1942–2006.

### Trend in menarcheal age among Indian women

**Figure 3:**
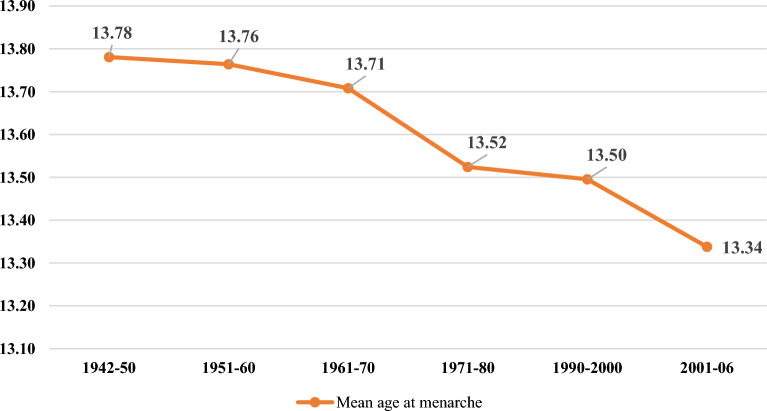
Trend in mean age at menarche among women in India.

Table 1 presents the mean age at menarche across birth cohorts among women in India. Overall, for women born during the 64-year period, the weighted mean age at menarche was 13.49 ± 1.21 years (95% CI: 13.49–13.50). Notably, the mean age at menarche showed a long-term declining trend towards the younger birth cohorts (Fig. 3). The mean age at menarche was reduced from 13.78 ± 1.40 to 13.34 ± 1.06 years, with a difference of about 0.44 years between the oldest and youngest cohorts in menarcheal age.

**Table 1:** Mean age at menarche by birth cohort among women in India.

Birth cohort	Mean	95% CI		SD	Percentage distribution of women	N
		LCI	UCI			
1942–50	13.78	13.76	13.81	1.40	2.3	13,851
1951–60	13.76	13.75	13.78	1.40	4.4	25,903
1961–70	13.71	13.69	13.72	1.35	6.0	34,972
1971–80	13.52	13.50	13.55	1.22	2.9	15,051
1990–2000	13.50	13.49	13.5	1.22	65.6	377,345
2001–06	13.34	13.33	13.34	1.06	18.9	111,668
Total	13.49	13.49	13.5	1.21	100	5,78,790

### Regional variations in menarcheal age among Indian women

Table 2 demonstrates the percentage distribution of menarcheal age across the regions of India. In regions like the North (35.1%), Central (36.9%) and South (31.1%), a majority of women had attained menarche at the age of 14 years. On the other hand, in the Eastern (44.4%) and Western (36.4%) parts of the country, the maximum number of women reported experiencing menarche at the age of 13 years. However, in the Northeastern region, nearly 36.1% of women reached menarche at an early age of 12 years.

**Table 2:** Distribution of age at menarche by regions of India.

Region	≤ 9	10	11	12	13	14	15	≥ 16
North	0.1	0.3	1.2	8.7	34.6	35.1	14.4	5.7
Central	0.1	0.2	0.8	8.2	34.2	36.9	14.7	5.0
East	0.2	0.6	2.0	18.9	44.4	24.7	6.7	2.4
Northeast	1.0	2.4	9.1	36.1	33.6	11.7	4.1	1.9
South	0.1	0.4	1.8	12.3	29.2	31.1	16.6	8.4
West	0.2	0.6	4.2	19.7	36.4	24.3	10.6	4.1
Total	0.2	0.5	2.2	14.3	36.3	29.9	12.0	4.7

**Figure 4:**
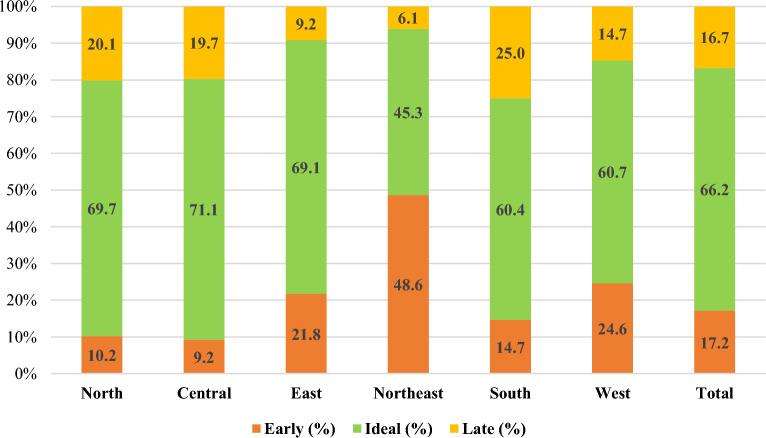
Distribution of age at menarche by regions of India.

Figure 4 presents the percentage distribution of age at menarche by regions of India. According to the results, in most of the regions, the percentage share for ideal menarche was highest. However, in the case of the Northeastern region, nearly 48.6% of women reported early menarche, whereas 45.3% of women reported the attainment of menarche between the ages of 13–14 years. Furthermore, in the Southern region, about one-fourth of women reported a late age at menarche. Likewise, nearly one-fifth of women residing in Northern India reported late menarche. Nevertheless, only 9.2% of women reported early menarche in Central India.

**Figure 5:**
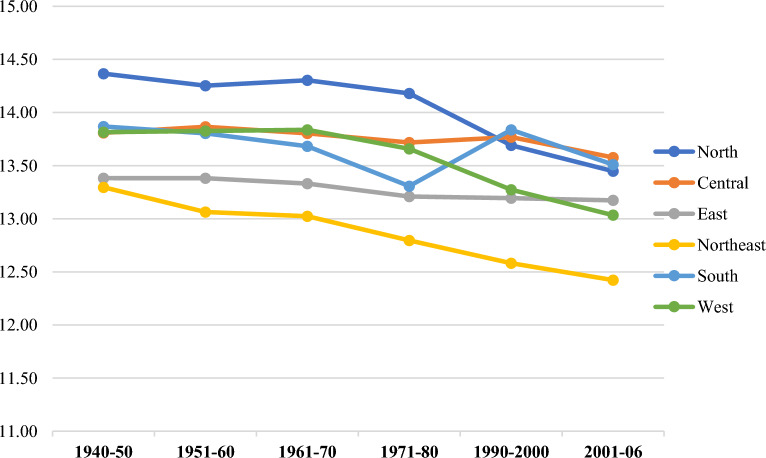
Birth cohort specific mean age at menarche among women across regions in India.

Figure 5 shows the regional heterogeneity in cohort-specific mean age at menarche among women in India born between 1942 and 2006. The mean age at menarche was highest in the Southern region i.e., 13.76 ± 1.34 years (95% CI: 13.75–13.77) followed by the Central (13.73 ± 1.10, 95% CI: 13.72–13.73) and Northern region (13.71 ± 1.15, 95% CI: 13.70–13.72) (Table 3). However, it was lowest in the Northeastern region, with a mean age at menarche of 12.62 ± 1.27 years (95% CI: 12.61–12.63). All the regions showed secular declining trends in menarcheal age except for the South and Central India. In addition, the estimates of mean age at menarche across geographic regions were compared by performing One-way ANOVA test and were found to be significantly different (p < 0.001).

**Table 3:** Mean age at menarche by regions of India.

Region	Mean	95% CI		SD	Percentage distribution of women	N
		LCI	UCI			
North	13.71	13.7	13.72	1.15	13.69	118,710
Central	13.73	13.72	13.73	1.10	27.17	155,452
East	13.21	13.2	13.21	1.09	23.5	104,101
Northeast	12.62	12.61	12.63	1.27	3.5	73,346
South	13.76	13.75	13.77	1.34	14.7	57,645
West	13.29	13.29	13.3	1.27	17.44	69,536
Total	13.49	13.49	13.5	1.21	100	578,790

### Association of socio-economic factors with menarcheal age

Table 4 demonstrates the distribution of the age at menarche and also presents a descriptive analysis of mean age at menarche across selected socio-economic variables. According to the distribution of the respondents' educational backgrounds, nearly 17% of the sampled women had no formal education. On the other hand, about 59% of women had completed their secondary education, whereas only 15% had higher education. As per the results, women with higher education attainment showed a later age at menarche (13.63; 95% CI: 13.62–13.63) than the others. Furthermore, in terms of wealth index, women who belonged to the poorest wealth quintile had the lowest mean age at menarche (13.41; 95% CI: 13.41–13.42). Moreover, among different caste groups, the lowest mean age at menarche was shown by ST women (13.38; 95% CI: 13.37–13.39), whereas women belonging to Other caste groups had the highest mean age at menarche (13.54; 95% CI:13.54–13.55). More than one-fourth of christian women (27.5%) reported an early attainment of menarche, whereas nearly 13% of muslim women reported experiencing later age at menarche. In addition, women belonged to the christian community had the lowest mean age at menarche (13.26; 95% CI: 13.24–13.27) as compared to the other religious groups. In terms of place of residence, urban women (13.47; 95%: 13.46–13.47) documented an earlier age at menarche than those who reside in rural areas (13.51; 95% CI: 13.50–13.51).

**Table 4:** Distribution of age at menarche and mean age at menarche by background characteristics.

Background characteristics	Age at menarche			Mean age	95% CI	p values	Percentage distribution of women	N
	Early (%)	Ideal (%)	Late (%)					
Education						p < 0.001		
No education	15.6	67.1	17.3	13.52	13.51–13.53		16.7	93,460
Primary	18.0	63.8	18.2	13.51	13.50–13.53		8.9	52,861
Secondary	18.2	66.3	15.5	13.45	13.45–13.45		59.0	351,370
Higher	14.3	65.9	19.8	13.63	13.62–13.63		15.4	80,827
Wealth index						p < 0.001		
Poorest	17.8	68.0	14.2	13.41	13.41–13.42		19.2	1,11,270
Poorer	18.1	66.6	15.3	13.44	13.43–13.45		21.3	1,23,104
Middle	17.2	65.4	17.5	13.51	13.51–13.52		21.1	1,22,110
Richer	16.5	64.8	18.6	13.55	13.55–13.56		20.3	1,17,735
Richest	16.0	66.1	17.9	13.55	13.55–13.56		18.1	1,04,571
Caste						p < 0.001		
SC	17.6	66.5	15.9	13.46	13.46–13.47		21.2	105,921
ST	20.3	65.2	14.5	13.38	13.37–13.39		9.8	99,401
Others	15.9	66.5	17.6	13.54	13.54–13.55		69.0	351,811
Religion						p < 0.001		
Hindu	16.3	66.4	17.3	13.50	13.49–13.50		69.2	373,316
Muslim	20.9	66.4	12.7	13.31	13.31–13.32		13.5	77,915
Christian	27.5	57.9	14.7	13.26	13.24–13.27		2.0	35,209
Others	14.1	64.7	21.2	13.69	13.69–13.70		15.3	92,350
Place of residence						p < 0.001		
Urban	18.1	65.9	16.0	13.47	13.46–13.47		30.0	150,554
Rural	16.8	66.3	17.0	13.51	13.50–13.51		70.0	428,236
Total	17.2	66.2	16.7	13.49	13.49–13.50		100	578,790

According to Supplementary Fig. S1, women with secondary education showed the highest mean age at menarche in earlier birth cohorts. However, in the case of later birth cohorts, it was highest for higher-educated women. Likewise, in the case of women born before the year 2000, those who belonged to higher economic status groups showed the highest mean age at menarche, whereas in the case of women born after 2000, the richest women showed the lowest mean (Supplementary Fig. S2). Among different caste groups, ST women demonstrated the lowest mean age at menarche in every birth cohort. On the other hand, the highest mean age at menarche was shown by the women belonged to the Others caste groups (Supplementary Fig. S3). Furthermore, Christian women used to show the highest mean age at menarche untill 1980. However, this is not the case for women born after the year 1990 (Supplementary Fig. S4). Considering place of residence, among women born before the year 1980, the mean age at menarche was found to be highest among urban women. However, for the later birth cohorts, rural dwellers reported the highest mean (Supplementary Fig. S5).

The result of the multiple linear regression model is presented in Table 5. After adjusting for socio-economic characteristics such as birth cohort, education, wealth index, caste, religion, place of residence, region, etc., the results demonstrated a significant negative association between the birth cohort and the menarcheal age of women. This confirmed the secular declining trend in age at menarche among Indian women. The results also suggested a significant positive association between educational attainment and age at menarche. Women with higher education had 0.211 year higher age at menarche than illiterate women. Furthermore, women from the richest wealth quintile were likely to experience their menarche 0.022 year earlier as compared to women from the poorest wealth quintile. Likewise, ST women were likely to experience their menarche 0.051 year earlier than the SC women. However, according to the results, women belonged to Others caste groups experienced menarche 0.068 year later than the SC women. The study also found statistically significant differences in mean menarcheal age across religions. Muslim women experienced menarche 0.162 year earlier than their hindu counterparts whereas, christian women were more likely to attain menarche 0.441 year later than hindu women. Moreover, women from rural areas were more likely to experience menarche 0.073 year later than their urban counterparts. The results also suggest statistically significant differences in menarcheal age among women across various regions of India.

**Table 5:** Association between background characteristics and age at menarche: result from multiple linear regression model.

Background characteristics	Coefficient						Total	95% CI	
	North	Central	East	Northeast	South	West			
Birth cohort									
1940–50^®^									
1951–60	−0.087**	0.031	0.021	−0.193***	−0.092**	−0.024	−0.039**	−0.065	−0.012
1961–70	−0.046	−0.008	−0.020	−0.300***	−0.239***	−0.035	−0.083***	−0.108	−0.057
1971–80	−0.165***	−0.133***	−0.141***	−0.532***	−0.599***	−0.210***	−0.272***	−0.303	−0.242
1990–2000	−0.652***	−0.249***	−0.107***	−0.851***	−0.244***	−0.866***	−0.431***	−0.454	−0.408
2001–06	−0.918***	−0.437***	−0.152***	−1.040***	−0.577***	−1.119***	−0.627***	−0.651	−0.603
Education									
No education^®^									
Primary	0.074***	0.099***	0.031*	0.147***	0.303***	0.062*	0.109***	0.096	0.123
Secondary	0.034**	0.070***	0.021*	0.119***	0.372***	0.349***	0.106***	0.095	0.117
Higher	0.119***	0.201***	0.132***	0.148***	0.477***	0.505***	0.211***	0.197	0.225
Wealth index									
Poorest^®^									
Poorer	0.018	0.003***	−0.032***	0.015	0.014	0.088**	0.004	−0.007	0.014
Middle	0.051**	0.010*	−0.023*	0.059**	0.091***	0.105***	0.046***	0.035	0.056
Richer	0.072***	0.019**	−0.041**	0.024	0.107***	0.058*	0.057***	0.045	0.069
Richest	0.021	−0.031***	−0.126***	−0.049	−0.006	−0.061*	−0.022*	−0.035	−0.008
Caste									
SC^®^									
ST	−0.059***	−0.244***	−0.092***	0.021	−0.057*	0.005	−0.051***	−0.063	−0.039
OBC/others	0.071***	0.035***	0.160***	0.011	0.104***	0.029*	0.068***	0.060	0.077
Religion									
Hindu^®^									
Muslim	−0.417***	−0.081***	−0.083***	0.032	−0.100***	−0.144***	−0.162***	−0.172	−0.152
Christian	−0.045	−0.188**	−0.062*	0.751***	0.208***	−0.287***	0.441***	0.424	0.457
Others	−0.005	0.108**	−0.026	0.458***	0.070*	−0.147*	0.116***	0.100	0.132
Place of residence									
Urban^®^									
Rural	0.160***	0.074***	0.090***	−0.097***	0.118***	0.066***	0.073***	0.064	0.081
Region									
North^®^									
Central	–	–	–	–	–	–	−0.005	−0.015	0.005
East	–	–	–	–	–	–	−0.509***	−0.520	−0.498
Northeast	–	–	–	–	–	–	−0.898***	−0.912	−0.884
South	–	–	–	–	–	–	−0.015*	−0.028	−0.002
West	–	–	–	–	–	–	−0.448***	−0.459	−0.436

## Discussion

In the present study, the majority of the women had attained menarche between the ages of 13–14 years (66.2%), with a mean age at menarche of 13.49 ± 1.21 years. In addition, nearly 17.2 percent of women had experienced an early age at menarche, whereas 16.7% had later age at menarche. Several studies from various nations have updated the mean menarcheal age during the past few decades and indicated a range of 12.0 to 13.0 years^32–34^. Moreover, for the past few decades, the mean age at menarche in various populations in India has varied from 15.2 to 12.5 years^24,25,35,36^. In addition, the secular trend for the decline in the mean age of menarche has been documented in some of the states^12,24,25^. However, the mean age at menarche observed in the current study is comparable to the findings of Pathak et al. (2014)^26^.

The present study also revealed a continuous secular decline in age at menarche through the birth years of 1942 to 2006 among Indian women. The mean menarcheal age was reduced from 13.78 ± 1.40 to 13.34 ± 1.06 years during the 64-year period. According to prior research, the decreased trend of menarcheal age was common around the world; however, the age at menarche varied^37–39^. Although the secular trend of age at menarche has lately slowed in countries like the Netherlands, Japan, Germany and Bulgaria and stayed steady in Belgium and Norway, the trend in China has persisted downward^40–42^. Furthermore, a previous study among Indian women using Indian Human Development Survey (IHDS) data also suggested a secular decline in age at menarche with a reduction of nearly one month per decade (Pathak et al., 2014)^26^.

A significant heterogeneity in mean menarcheal age among Indian women across geographical regions was observed over time. Women from the Northeastern region of India experienced menarche at an early age as compared to their counterparts. This is in line with the study by Pathak et al. (2014)^26^, which recorded the lowest mean age at menarche in the Northeastern states of Assam, Arunachal Pradesh and Sikkim etc. On the other hand, the Southern region of India reported the highest mean age at menarche. The disparities in mean menarcheal age can be attributed to the differences in the environment, climate and food habits in different regions of the country^43–45^.

Furthermore, the present study demonstrates significant socioeconomic patterning of the mean age at menarche among Indian women. Interestingly, compared to their counterparts, women from economically sound families had a much lower mean age at menarche. Several studies have demonstrated that socioeconomic factors have an impact on the menarcheal onset, with girls growing up in more deprived situations experiencing later menarche as they are unable to get the nutrients they need for proper growth and development in order to attain menarche^28,30,46^. Moreover, in the current study, a relatively later age at menarche was reported among women residing in rural areas as compared to urban dwellers.

### Strength and limitation

The present study is based on the data from a large-scale, nationally representative survey in India; therefore, one of the major strengths is the wider relevance of its results. In addition, although previous research on age at menarche^26^ indicated a declining trend among birth cohorts born between 1950 and 1990, an update on the menarcheal age of cohorts born after 1990 is essential to verify the secular decline in the nation. The menarcheal age of women born between 1942 and 2006 has been examined in the current study. Therefore, this research can be considered as an expansion of the previous one. Furthermore, the distribution of early, ideal and late age at menarche as well as the regional variations in the menarcheal ages among Indian women have been demonstrated by this study. Although the present study contributes to the literature on menarche, there are certain limitations that must be taken into account while interpreting the results. Firstly, the recall method is used to calculate the menarcheal age. The recall approach has come under criticism from certain research for overreporting menarcheal age, while other studies have accused it of underreporting menarcheal age^47^. However, some have compared recall data with information from other sources, noting that the recall approach yields estimates that are generally reliable^48^. Secondly, this study utilized NFHS data sets, which provide retrospective information on menarcheal age. Therefore, any estimations derived using the current data will give older reference dates than those found in the most recent status quo research. However, to address this problem, the study participants were grouped according to their year of birth. Thus, the comparative pseudo-cohort technique became apparent as a possible strength of the current study. Furthermore, given the cross-sectional nature of the data, we were unable to establish any causal linkages between the outcome and exposure variables.

## Conclusion

In conclusion, a secular declining trend in the age at menarche was observed among women born from 1942 to 2006 in India. The study also indicated a significant variation in the mean age at menarche across birth cohorts of women, with the youngest cohort reporting the lowest mean age at menarche. It also highlighted significant socio-economic patterning in menarcheal age among women. Secular changes in age at menarche may have implications for women’s lifetime exposure to circulating endogenous hormones and subsequent risk of adverse health outcomes.

## Methods

### Data source

In this study, a birth cohort approach was used by polling data from the 1st (1992–93), 4th (2015–16) and 5th (2019–21) rounds of the NFHS. The total sample size was 578,790. The detailed sample selection procedure was explained in Supplementary Fig. S6. The second and third rounds of NFHS were not considered in the analysis, as no information on age at menarche was collected in these two rounds. The survey is conducted under the stewardship of the Ministry of Health and Family Welfare, Government of India and coordinated by the International Institute of Population Sciences, Mumbai. The International Institute for Population Sciences, being the nodal agency was responsible for obtaining the ethical approval for conducting and disseminating the data for the survey. Each successive round of the NFHS has had two specific goals: (a) to provide essential data on health and family welfare needed by the Ministry of Health and Family Welfare and other agencies for policy and programme purposes, and (b) to provide information on important emerging health and family welfare issues.

### Outcome variable

The outcome variable of this study was age at menarche. There was a direct question on age at menarche i.e. “How old were you when you had your first monthly period?” in the woman's questionnaire in NFHS-I, NFHS-IV and NFHS-V. In NFHS-I, this question was asked to all women respondents irrespective of their age. However, in NFHS-IV and NFHS-V, only women in the age group of 15–24 years were asked about the age at their first monthly period. This information was used to carry out this study.

*Age at menarche*: For some analysis the age at menarche was categorized into three groups viz. early menarche (less than 13 years of age), ideal menarche (13–14 years) and late menarche (more than 14 years of age)^30^.

### Covariates

The covariates used in the analyses were as follows:

- Birth cohort: the information on year of birth of women was used to identify the birth cohorts. It was categorised as: 1942–1950, 1951–1960, 1961–1970, 1971–1980, 1990–2000, 2001–2006. This study lacks information on the age at menarche for women born between 1981 and 1989 since no data on menarche was gathered during the second or third rounds of the NFHS.
- Education: this variable describes the educational level of the respondents. It was recoded as: ‘No education’, ‘Primary’, ‘Secondary’ and ‘Higher’.
- Wealth index: this variable represents the economic status of the household of the respondents. Scores were assigned to households depending on the amount and types of consumer goods they own, which can range from a television to a bicycle or a car, as well as home features such as water supply, toilet facilities, and flooring materials. Principal component analysis was used to calculate these scores. The national wealth quintiles were calculated by assigning a score to each typical household member, rating each individual in the household population according to their score, and then dividing the distribution into five equal groups, each having 20 percent of the population. It was recoded as: ‘Poorest’, ‘Poorer’, ‘Middle’, ‘Richer’, ‘Richest’.
- Caste: this variable was recoded into three categories, ‘Scheduled Caste’ (SC), ‘Scheduled Tribe’ (ST), and ‘Others’.
- Religion: respondents were asked about their religion and the responses were like ‘Hindu’, ‘Muslims’, ‘Christian’, ‘Sikh’, ‘Buddhist’, ‘Jain’, ‘Jewish’, ‘Parsi’, ‘No religion’ and ‘Others’. However, for the analysis, the variable was categorized into four groups in which three main religions such as ‘Hindu’, ‘Muslim’ and ‘Christian’ were considered in three separate groups and the rest were kept in one group recorded as ‘Other’.
- Place of residence: it was categorized into two groups; ‘Urban’ and ‘Rural’.
- Region: this variable represents various regions of India. The ‘state’ variable was categorized into six groups in order to form this variable. Jammu and Kashmir, Himachal Pradesh, Punjab, Chandigarh, Uttarakhand, Haryana, Delhi and Rajasthan make up the Northern region. Madhya Pradesh, Uttar Pradesh and Chhattisgarh make up the Central region. Bihar, Jharkhand, Odisha, and West Bengal make up the Eastern region. Arunachal Pradesh, Assam, Manipur, Meghalaya, Mizoram, Nagaland, Sikkim, and Tripura make up the North‐eastern area. Dadra & Nagar Haveli, Daman & Diu, Goa, Gujarat, and Maharashtra make up the Western area. Andhra Pradesh, Karnataka, Kerala, Puducherry, Tamil Nadu, Telangana, Lakshadweep and Andaman & Nicobar Islands make up the Southern region. These six groups were coded as: ‘North’, ‘Central’, ‘Northeast’, ‘East’, ‘South’, ‘West’.

### Statistical analysis

Descriptive statistics and bivariate analyses (crosstabs) were performed to present the distribution of age at menarche and mean age at menarche across birth cohorts, regions and other covariates such as education, wealth index, caste, religion and place of residence. Further, One-way analysis of variance (ANOVA) was used to test the statistical differences in mean age at menarche across categories of covariates. Considering the continuous nature of the outcome variable, a multiple linear regression model was fitted to examine the trend in age at menarche and further to investigate the association of covariates with menarcheal age among Indian women. The regression model was adjusted for covariates like birth cohort, education, wealth index, caste, religion, place of residence and region. A stepwise regression using a bidirectional elimination approach was performed to identify the covariates used for adjustment in the model. All the analyses were carried out using Stata version 14.

The multiple linear regression model can be written as follow:

$${\text{y}}_{{\text{i}}} = \, \beta_{0} + \, \beta_{{1}} {\text{x}}_{{{\text{i1}}}} + \, \beta_{{2}} {\text{x}}_{{{\text{i2}}}} + \cdots + \, \beta_{{\text{p}}} {\text{x}}_{{{\text{ip}}}} + \varepsilon$$

where, y_i_ is the dependent variable, x_i_ is the explanatory/independent variables, β_0_ is the y-intercept, β_p_ is the slope coefficients for each explanatory variable, ϵ is the error term.

### Supplementary Information

Supplementary Figures.

## Data Availability

The data used in this research is publicly available on DHS website. Any individual can register and easily obtained data in electronic version from the following website https://dhsprogram.com/data/new-user-registration.cfm.
