# Supplementary material for: Secular trend in age at menarche among Indian women
Source: Sci Rep. 2024 Mar 5;14:5398. doi: 10.1038/s41598-024-55657-7 (PMC10914750; doi:10.1038/s41598-024-55657-7)

**Figure S1:** **Birth cohort specific mean age at menarche by educational attainment**

**Figure S2:** **Birth cohort specific mean age at menarche by wealth index**

**Figure S3:** **Birth cohort specific mean age at menarche by caste**

**Figure S4:** **Birth cohort specific mean age at menarche by religion**

**Figure S5:** **Birth cohort specific mean age at menarche by place of residence**

**Figure S6: Flowchart of sample selection**


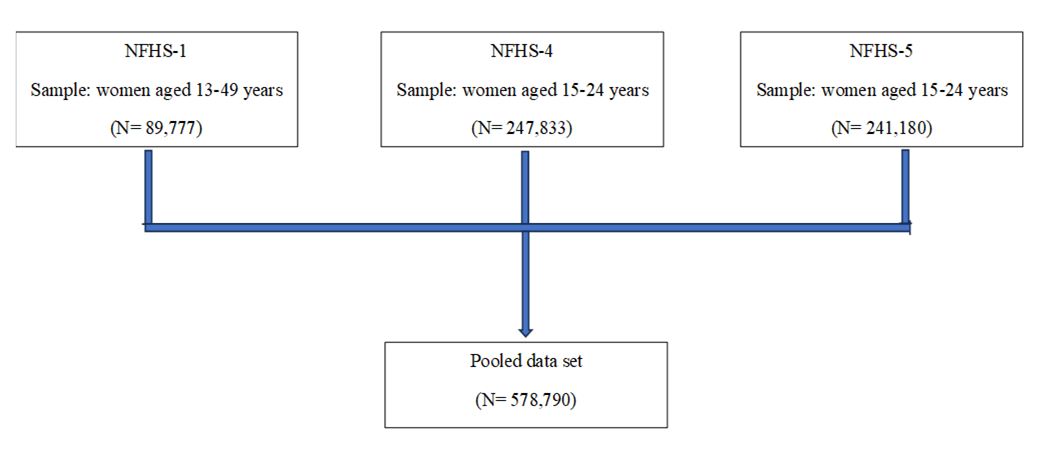

Supplement: Supplementary file 1 — Supplementary Figures. [file 41598_2024_55657_MOESM1_ESM.docx]
